# Supplementary material for: Multiple event segmentation mechanisms in the human brain
Source: eLife. 2026 Jul 7;14:RP107955. doi: 10.7554/eLife.107955 (PMC13341109; doi:10.7554/eLife.107955)
Supplement: Peer review history

# Editor report: eLife Assessment

**DOI:** 10.7554/eLife.107955.3.sa0

**Authors:** Jonathan Erik Peelle

**Affiliations:** Northeastern University United States

**Keywords:** Solid, Valuable

This **valuable** study tests whether prediction error or prediction uncertainty controls how the brain segments continuous experience into events. The paper uses validated models that predict human behavior to analyze multivariate neural pattern changes during naturalistic movie watching. The authors provide **solid** evidence that there are overlapping but partially distinct brain dynamics for each signal.

# Referee report: Reviewer #1 (Public review)

**DOI:** 10.7554/eLife.107955.3.sa1

Summary:

This paper investigates the control signals that drive event model updating during continuous experience. The authors apply predictions from previously published computational models to fMRI data acquired while participants watched naturalistic video stimuli. They first examine the time course of BOLD pattern changes around human-annotated event boundaries, revealing pattern changes preceding the boundary in anterior temporal and then parietal regions, followed by pattern stabilization across many regions. The authors then analyze time courses around boundaries generated by a model that updates event models based on prediction error and another that uses prediction uncertainty. These analyses reveal overlapping but partially distinct dynamics for each boundary type, suggesting that both signals may contribute to event segmentation processes in the brain.

Strengths:

The question addressed by this paper is of high interest to researchers working on event cognition, perception, and memory. There has been considerable debate about what kinds of signals drive event boundaries, and this paper directly engages with that debate by comparing prediction error and prediction uncertainty as candidate control signals.

The authors use computational models that explain significant variance in human boundary judgments, and they report the variance explained clearly in the paper.

The authors' method of using computational models to generate predictions about when event model updating should occur is a valuable mechanistic alternative to methods like HMM or GSBS, which are data-driven.

The paper utilizes an analysis framework that characterizes how multivariate BOLD pattern dissimilarity evolves before and after boundaries. This approach offers an advance over previous work focused on just the boundary or post-boundary points.

Weaknesses:

Boundaries derived from prediction error and uncertainty are correlated for the naturalistic stimuli. This raises some concerns about how well their distinct contributions to brain activity can be separated. While the authors attempt to look at the unique variance, there is a limit to how effectively this can be done without experimentally dissociating prediction error and uncertainty.

The authors reports an average event length of ~20 seconds, and they also look +20 and -20 seconds around each event boundary. Thus, it's unclear how often pre- and post-boundary timepoints are part of adjacent events. This complicates the interpretations of the reported timecourses.

# Referee report: Reviewer #2 (Public review)

**DOI:** 10.7554/eLife.107955.3.sa2

Summary:

Tan et al. examined how multivoxel patterns shift in time windows surrounding event boundaries caused by both prediction errors and prediction uncertainty. They observed that some regions of the brain show earlier pattern shifts than others, followed by periods of increased stability. The authors combine their recent computational model to estimate event boundaries that are based on prediction error vs. uncertainty and use this to examine the moment-to-moment dynamics of pattern changes. I believe this is a meaningful contribution that will be of interest to memory, attention, and complex cognition research.

Strengths:

The authors have shown exceptional transparency in terms of sharing their data, code, and stimuli which is beneficial to the field for future examinations and to the reproduction of findings. The manuscript is well written with clear figures. The study starts from a strong theoretical background to understand how the brain represents events and have used a well-curated set of stimuli. Overall, the authors extend the event segmentation theory beyond prediction error to include prediction uncertainty which is an important theoretical shift that has implications in episodic memory encoding, use of semantic and schematic knowledge and to attentional processing.

Weaknesses:

(1) I am not fully satisfied with the author's explanation of pattern shifts occurring 11.9s prior to event boundaries. The average length of time for an event was 21.4 seconds. The window around the identified event boundaries was 20 seconds on either side. The earliest identified pattern shift peaks occur at 11.9s prior to the actual event boundary. This would mean on average, a pattern shift is occurring approximately at the midway point of the event (11.9s prior to a boundary of a 21.4s event is approx. the middle of an event). The authors offer up an explanation in which top down regions signal an update that propagates to lower order regions closer to the boundary. To make this interpretation concrete, they added an example: "in a narrative where a goal is reached midway-for instance, a mystery solved before the story formally ends-higher-order regions may update the event representation at that point, and this updated model then cascades down to shape processing in lower-level regions". This might make sense in a one-off case of irregular storytelling, but it is odd to think this would generalize. If an event is occurring and a given collection of regions represent that event, it doesn't follow the accepted convention of multivariate representational analysis that that set of regions would undergo such a large shift in patterns in the middle of an event. The stabilization of these patterns taking so long is also odd to me. I suspect some of these findings may be due to the stimuli used in this experiment and I am not confident this would generalize and invite the authors to disagree and explain. In the case of the exercise routine video, I try to imagine going from the push-up event to the jumping jack event. The actor stops doing pushups, stands up, and moves minimally for 16 seconds (these lulls are not uncommon). At that point they start doing jumping jacks. It is immediately evident from that moment on that jumping jacks will be the kind of event you are perceiving which may explain the long delay in event pattern stabilisation. Then about 11.9s prior to the end of the event, when the person is still performing jumping jacks (at this point they have been performing jumping jacks for 6 seconds), I would expect the brain to still be expecting this " jumping jacks event". For some reason at this point multivariate patterns in higher order regions shift. I do not understand what kind of top down processing is happening here and the reviewers need to be more concrete in their explanation because as of right now it is ill-defined. I also recognize that being specific to jumping jacks is maybe unfair, but this would apply to the push-ups, granola bar eating, or table cleaning events in the same manner. I suspect one possibility is that the participants realize that the stereotyped action of jumping jacks is going to continue and, thus, mindwander to other thoughts while waiting for novel, informative information to be presented. This explanation would challenge the more active top down processing assumed by the authors.

I had provided a set of concerns to the authors that were not part of the public review and were not addressed. I was unaware of the exact format of the eLife approach, but I think they are worth open discussion so I am adding them here for consideration. Apologies for any confusion.

(2) Why did the authors not examine event boundary activity magnitude differences from the uncertainty vs error boundaries? I see that the authors have provided the data on the openneuro. However, it seems like the difference in activity maps would not only provide extra contextualization of the findings, but also be fairly trivial. Just by eye-balling the plots, it appears as though there may be activity differences in the mPFC occurring shortly after a boundary between the two. Given this regions role in prediction error and schema, it would be important to understand whether this difference is merely due to thresholding effects or is statistically meaningful.

(3) Further, the authors omitted all subcortical regions some of which would be especially interesting such as the hippocampus, basal ganglia, ventral tegmental area. These regions have a rich and deep background in event boundary activity, and prediction error. Univariate effects in these regions may provide interesting effects that might contextualize some of the pattern shifts in the cortex.

(3) I see that field maps were collected, but the fmriprep methods state that susceptibility distortion correction was not performed. Is there a reason to omit this?

(4) How many events were present in the stimuli?

# Referee report: Reviewer #3 (Public review)

**DOI:** 10.7554/eLife.107955.3.sa3

Summary:

The aim of this study was to investigate the temporal progression of the neural response to event boundaries in relation to uncertainty and error. Specifically, the authors asked 1. How neural activity changes before and after event boundaries 2. If uncertainty and error both contribute to explaining the occurrence of event boundaries and 3. If uncertainty and error have unique contributions to explaining the temporal progression of neural activity.

Strengths:

One strength of this paper is that it builds on an already validated computational model. It relies on straightforward and interpretable analysis techniques to answer the main question, with a smart combination of pattern similarity metrics and FIR. This combination of methods may also be an inspiration to other researchers in the field working on similar questions. The paper is well written and easy to follow. The paper convincingly shows that 1. There is a temporal progression of neural activity change before and after an event boundary 2. Event boundaries are predicted best by the combination of uncertainty and error signals.

Weaknesses:

Regarding question 3, the results are less convincing. Although the analyses in Figure S1 show that there are some unique contributions of uncertainty and error, it is unclear to what extent the results in Figure 7 are driven by shared variance. Therefore, it is not clear to what extent the main claim in the abstract is due to shared or unique variance. More specific comments are provided below.

The other issue is the distance between events is short compared to the pre-onset effects that are observed. Halfway the distance between two events there are already neural signatures of change relating to the upcoming event boundary. I wonder if methodological issues could explain this effect and if not, what could allow participants to notice the impending event boundary.

Impact:

If these comments can be addressed sufficiently, I expect that this work will impact the field in its thinking on what drives event boundaries and spur interest in understanding the mechanisms behind the temporal progression of neural activity around these boundaries.

Comments

(1) The correlation between uncertainly and prediction error is very high, which makes it challenging to disentangle the effects of both on the neural response. The analysis in Figure S1 shows that the two predictors indeed have dissociable contributions. However, the results mainly reported in the discussion section and abstract still rely on models where only one of these factors is included at a time. This makes it debatable whether these specific networks mentioned really reflect unique contributions of each of these components. I specifically refer to this statement in the abstract: "Error-driven boundaries were associated with early pattern shifts in ventrolateral prefrontal areas, followed by pattern stabilization in prefrontal and temporal areas. Uncertainty-driven boundaries were linked to shifts in parietal regions within the dorsal attention network, with minimal subsequent stabilization. ". I would encourage repeating all analyses (also the ones in figure 7) with a models that includes both predictors and showing both results in the manuscript, so it is clear which regions really show unique variance related to one of the predictors. I also wonder why it is necessary to look at model comparisons between the combined and unique models, rather than simply reporting the significance of each predictor in the combined model.

(2) The distance between event boundaries ranges between 20 and 30 seconds. The early pre-boundary effect that are observed in the manuscript occur at -12 seconds. This means that these effects occur roughly halfway between the previous and current event. This seems much earlier than expected. That is why I worry that the FIR analyses might not be able to distinguish effects of the previous event from effects of the upcoming event. What evidence is there that the FIR analyses can actually properly show the return to baseline? One way to address this might be to randomize the locations of the event boundaries while preserving the distance between them and rerun the models. This will give a null-model with the same event distances and should be able to distinguish this temporal overlap from the true effects of event boundaries.

(3) If the analyses in point 2 confirm that there is indeed an event-boundary related change that occurs 12 seconds before event onset, it is important to consider what might cause these changes. Are there cues in the movie that indicate that an event boundary is coming? It would be interesting to investigate whether uncertainty and error are higher than expected at 12 seconds pre-onset.

# Author comment: Author response

**DOI:** 10.7554/eLife.107955.3.sa4

**Authors:** Tan T Nguyen, Jo A Etzel, Matthew A Bezdek, Jeffrey Zacks

**Affiliations:** Washington University in St. Louis Saint Louis United States; Washington University in St. Louis Saint Louis United States; Washington University in St. Louis Saint Louis United States; Washington University in St. Louis St. Louis United States

The following is the authors’ response to the original reviews.

**Public Reviews:**

**Reviewer #1 (Public review):**

Summary:

This paper investigates the control signals that drive event model updating during continuous experience. The authors apply predictions from previously published computational models to fMRI data acquired while participants watched naturalistic video stimuli. They first examine the time course of BOLD pattern changes around human-annotated event boundaries, revealing pattern changes preceding the boundary in anterior temporal and then parietal regions, followed by pattern stabilization across many regions. The authors then analyze time courses around boundaries generated by a model that updates event models based on prediction error and another that uses prediction uncertainty. These analyses reveal overlapping but partially distinct dynamics for each boundary type, suggesting that both signals may contribute to event segmentation processes in the brain.

Strengths:

(1) The question addressed by this paper is of high interest to researchers working on event cognition, perception, and memory. There has been considerable debate about what kinds of signals drive event boundaries, and this paper directly engages with that debate by comparing prediction error and prediction uncertainty as candidate control signals.

(2) The authors use computational models that explain significant variance in human boundary judgments, and they report the variance explained clearly in the paper.

(3) The authors' method of using computational models to generate predictions about when event model updating should occur is a valuable mechanistic alternative to methods like HMM or GSBS, which are data-driven.

(4) The paper utilizes an analysis framework that characterizes how multivariate BOLD pattern dissimilarity evolves before and after boundaries. This approach offers an advance over previous work focused on just the boundary or post-boundary points.

We appreciate this reviewer’s recognition of the significance of this research problem, and of the value of the approach taken by this paper.

Weaknesses:

(1) While the paper raises the possibility that both prediction error and uncertainty could serve as control signals, it does not offer a strong theoretical rationale for why the brain would benefit from multiple (empirically correlated) signals. What distinct advantages do these signals provide? This may be discussed in the authors' prior modeling work, but is left too implicit in this paper.

We added a brief discussion in the introduction highlighting the complementary advantages of prediction error and prediction uncertainty, and cited prior theoretical work that elaborates on this point. Specifically, we now note that prediction error can act as a reactive trigger, signaling when the current event model is no longer sufficient (Zacks et al., 2007). In contrast, prediction uncertainty is framed as proactive, allowing the system to prepare for upcoming changes even before they occur (Baldwin & Kosie, 2021; Kuperberg, 2021). Together, this makes clearer why these two signals could each provide complementary benefits for effective event model updating.

"One potential signal to control event model updating is prediction error—the difference between the system’s prediction and what actually occurs. A transient increase in prediction error is a valid indicator that the current model no longer adequately captures the current activity. Event Segmentation Theory (EST; Zacks et al., 2007) proposes that event models are updated when prediction error increases beyond a threshold, indicating that the current model no longer adequately captures ongoing activity. A related but computationally distinct proposal is that prediction uncertainty (also termed "unpredictability") can serve as a control signal (Baldwin & Kosie, 2021). The advantage of relying on prediction uncertainty to detect event boundaries is that it is inherently proactive: the cognitive system can start looking for cues about what might come next before the next event starts (Baldwin & Kosie, 2021; Kuperberg, 2021). "

(2) Boundaries derived from prediction error and uncertainty are correlated for the naturalistic stimuli. This raises some concerns about how well their distinct contributions to brain activity can be separated. The authors should consider whether they can leverage timepoints where the models make different predictions to make a stronger case for brain regions that are responsive to one vs the other.

We addressed this concern by adding an analysis that explicitly tests the unique contributions of prediction error– and prediction uncertainty–driven boundaries to neural pattern shifts. In the revised manuscript, we describe how we fit a combined FIR model that included both boundary types as predictors and then compared this model against versions with only one predictor. This allowed us to identify the variance explained by each boundary type over and above the other. The results revealed two partially dissociable sets of brain regions sensitive to error- versus uncertainty-driven boundaries (see Figure S1), strengthening our argument that these signals make distinct contributions.

"To account for the correlation between uncertainty-driven boundaries and error-driven boundaries, we also fitted a FIR model that predicted pattern dissimilarity from both types of boundaries (combined FIR) for each parcel. Then, we performed two likelihood ratio tests: combined FIR to error FIR, which measures the unique contribution of uncertainty boundaries to pattern dissimilarity, and combined FIR to uncertainty FIR, which measures the unique contribution of error boundaries to pattern dissimilarity. The analysis also revealed two dissociable sets of brain regions associated with each boundary type (see Figure S1)."

(3) The authors refer to a baseline measure of pattern dissimilarity, which their dissimilarity measure of interest is relative to, but it's not clear how this baseline is computed. Since the interpretation of increases or decreases in dissimilarity depends on this reference point, more clarity is needed.

We clarified how the FIR baseline is estimated in the methods section. Specifically, we now explain that the FIR coefficients should be interpreted relative to a reference level, which reflects the expected dissimilarity when timepoints are far from an event boundary. This makes it clear what serves as the comparison point for observed increases or decreases in dissimilarity.

"The coefficients from the FIR model indicate changes relative to baseline, which can be conceptualized as the expected value when far from event boundaries."

(4) The authors report an average event length of ~20 seconds, and they also look at +20 and -20 seconds around each event boundary. Thus, it's unclear how often pre- and post-boundary timepoints are part of adjacent events. This complicates the interpretations of the reported time courses.

This is related to reviewer's 2 comment, and it will be addressed below.

(5) The authors describe a sequence of neural pattern shifts during each type of boundary, but offer little setup of what pattern shifts we might expect or why. They also offer little discussion of what cognitive processes these shifts might reflect. The paper would benefit from a more thorough setup for the neural results and a discussion that comments on how the results inform our understanding of what these brain regions contribute to event models.

We thank the reviewer for this advice on how better to set the context for the different potential outcomes of the study. We expanded both the introduction and discussion to better set up expectations for neural pattern shifts and to interpret what these shifts may reflect. In the introduction, we now describe prior findings showing that sensory regions tend to update more quickly than higher-order multimodal regions (Baldassano et al., 2017; Geerligs et al., 2021, 2022), and we highlight that it remains unclear whether higher-order updates precede or follow those in lower-order regions. We also note that our analytic approach is well-suited to address this open question. In the discussion, we then interpret our results in light of this framework. Specifically, we describe how we observed early shifts in higher-order areas such as anterior temporal and prefrontal cortex, followed by shifts in parietal and dorsal attention regions closer to event boundaries. This pattern runs counter to the traditional bottom-up temporal hierarchy view and instead supports a model of top-down updating, where high-level representations are updated first and subsequently influence lower-level processing (Friston, 2005; Kuperberg, 2021). To make this interpretation concrete, we added an example: in a narrative where a goal is reached midway—for instance, a mystery solved before the story formally ends—higher-order regions may update the event representation at that point, and this updated model then cascades down to shape processing in lower-level regions. Finally, we note that the widespread stabilization of neural patterns after boundaries may signal the establishment of a new event model.

Excerpt from Introduction:

“More recently, multivariate approaches have provided insights into neural representations during event segmentation. One prominent approach uses hidden Markov models (HMMs) to detect moments when the brain switches from one stable activity pattern to another (Baldassano et al., 2017) during movie viewing; these periods of relative stability were referred to as "neural states" to distinguish them from subjectively perceived events. Sensory regions like visual and auditory cortex showed faster transitions between neural states. Multi-modal regions like the posterior medial cortex, angular gyrus, and intraparietal sulcus showed slower neural state shifts, and these shifts aligned with subjectively reported event boundaries. Geerligs et al. (2021, 2022) employed a different analytical approach called Greedy State Boundary Search (GSBS) to identify neural state boundaries. Their findings echoed the HMM results: short-lived neural states were observed in early sensory areas (visual, auditory, and somatosensory cortex), while longer-lasting states appeared in multi-modal regions, including the angular gyrus, posterior middle/inferior temporal cortex, precuneus, anterior temporal pole, and anterior insula. Particularly prolonged states were found in higher-order regions such as lateral and medial prefrontal cortex.

The previous evidence about evoked responses at event boundaries indicates that these are dynamic phenomena evolving over many seconds, with different brain areas showing different dynamics (Ben-Yakov & Henson, 2018; Burunat et al., 2024; Kurby & Zacks, 2018; Speer et al., 2007; Zacks, 2010). Less is known about the dynamics of pattern shifts at event boundaries (e.g. whether shifts observed in higher-order regions precedes or follow shifts observed in lower-level regions), because the HMM and GSBS analysis methods do not directly provide moment-by-moment measures of pattern shifts. Both the spatial and temporal aspects of evoked responses and pattern shifts at event boundaries have the potential to provide evidence about two potential control processes (error-driven and uncertainty-driven) for event model updating.”

Excerpt from Discussion:

“We first characterized the neural signatures of human event segmentation by examining both univariate activity changes and multivariate pattern changes around subjectively identified event boundaries. Using multivariate pattern dissimilarity, we observed a structured progression of neural reconfiguration surrounding human-identified event boundaries. The largest pattern shifts were observed near event boundaries (~4.5s before) in dorsal attention and parietal regions; these correspond with regions identified by Geerligs et. al as shifting their patterns on a fast to intermediate timescale (2022). We also observed smaller pattern shifts roughly 12 seconds prior to event boundaries in higher-order regions within anterior temporal cortex and prefrontal cortex, and these are slow-changing regions identified by Geerligs et. al (2022). This is puzzling. One prevalent proposal, based on the idea of a cortical hierarchy of increasing temporal receptive windows (TRWs), suggests that higher-order regions should update representations after lower-order regions do (Chang et al., 2021). In this view, areas with shorter TRWs (e.g., word-level processors) pass information upward, where it is integrated into progressively larger narrative units (phrases, sentences, events). This proposal predicts neural shifts in higher-order regions to follow those in lower-order regions. By contrast, our findings indicate the opposite sequence. Our findings suggest that the brain might engage in top-down event representation updating, with changes in coarser-grain representations propagating downward to influence finer-grain representations. (Friston, 2005; Kuperberg, 2021). For example, in a narrative where the main goal is achieved midway—such as a detective solving a mystery before the story formally ends—higher-order regions might update the overarching event representation at that point, and this updated model could then cascade down to reconfigure how lower-level regions process the remaining sensory and contextual details. In the period after a boundary (around +12 seconds), we found widespread stabilization of neural patterns across the brain, suggesting the establishment of a new event model. Future work could focus on understanding the mechanisms behind the temporal progression of neural pattern changes around event boundaries.”

**Reviewer #2 (Public review):**

Summary:

Tan et al. examined how multivoxel patterns shift in time windows surrounding event boundaries caused by both prediction errors and prediction uncertainty. They observed that some regions of the brain show earlier pattern shifts than others, followed by periods of increased stability. The authors combine their recent computational model to estimate event boundaries that are based on prediction error vs. uncertainty and use this to examine the moment-to-moment dynamics of pattern changes. I believe this is a meaningful contribution that will be of interest to memory, attention, and complex cognition research.

Strengths:

The authors have shown exceptional transparency in terms of sharing their data, code, and stimuli, which is beneficial to the field for future examinations and to the reproduction of findings. The manuscript is well written with clear figures. The study starts from a strong theoretical background to understand how the brain represents events and has used a well-curated set of stimuli. Overall, the authors extend the event segmentation theory beyond prediction error to include prediction uncertainty, which is an important theoretical shift that has implications in episodic memory encoding, the use of semantic and schematic knowledge, and attentional processing.

We thank the reader for their support for our use of open science practices, and for their appreciation of the importance of incorporating prediction uncertainty into models of event comprehension.

Weaknesses:

The data presented is limited to the cortex, and subcortical contributions would be interesting to explore. Further, the temporal window around event boundaries of 20 seconds is approximately the length of the average event (21.4 seconds), and many of the observed pattern effects occur relatively distal from event boundaries themselves, which makes the link to the theoretical background challenging. Finally, while multivariate pattern shifts were examined at event boundaries related to either prediction error or prediction uncertainty, there was no exploration of univariate activity differences between these two different types of boundaries, which would be valuable.

The fact that we observed neural pattern shifts well before boundaries was indeed unexpected, and we now offer a more extensive interpretation in the discussion section. Specifically, we added text noting that shifts emerged in higher-order anterior temporal and prefrontal regions roughly 12 seconds before boundaries, whereas shifts occurred in lower-level dorsal attention and parietal regions closer to boundaries. This sequence contrasts with the traditional bottom-up temporal hierarchy view and instead suggests a possible top-down updating mechanism, in which higher-order representations reorganize first and propagate changes to lower-level areas (Friston, 2005; Kuperberg, 2021). (See excerpt for Reviewer 1’s comment #5.)

With respect to univariate activity, we did not find strong differences between error-driven and uncertainty-driven boundaries. This makes the multivariate analyses particularly informative for detecting differences in neural pattern dynamics. To support further exploration, we have also shared the temporal progression of univariate BOLD responses on OpenNeuro (BOLD_coefficients_brain_animation_pe_SEM_bold.html and BOLD_coefficients_brain_animation_uncertainty_SEM_bold.html in the derivatives/figures/brain_maps_and_timecourses/ directory; https://doi.org/10.18112/openneuro.ds005551.v1.0.4) for interested researchers.

**Reviewer #3 (Public review):**

Summary:

The aim of this study was to investigate the temporal progression of the neural response to event boundaries in relation to uncertainty and error. Specifically, the authors asked (1) how neural activity changes before and after event boundaries, (2) if uncertainty and error both contribute to explaining the occurrence of event boundaries, and (3) if uncertainty and error have unique contributions to explaining the temporal progression of neural activity.

Strengths:

One strength of this paper is that it builds on an already validated computational model. It relies on straightforward and interpretable analysis techniques to answer the main question, with a smart combination of pattern similarity metrics and FIR. This combination of methods may also be an inspiration to other researchers in the field working on similar questions. The paper is well written and easy to follow. The paper convincingly shows that (1) there is a temporal progression of neural activity change before and after an event boundary, and (2) event boundaries are predicted best by the combination of uncertainty and error signals.

We thank the reviewer for their thoughtful and supportive comments, particularly regarding the use of the computational model and the analysis approaches.

Weaknesses:

(1) The current analysis of the neural data does not convincingly show that uncertainty and prediction error both contribute to the neural responses. As both terms are modelled in separate FIR models, it may be that the responses we see for both are mostly driven by shared variance. Given that the correlation between the two is very high (r=0.49), this seems likely. The strong overlap in the neural responses elicited by both, as shown in Figure 6, also suggests that what we see may mainly be shared variance. To improve the interpretability of these effects, I think it is essential to know whether uncertainty and error explain similar or unique parts of the variance. The observation that they have distinct temporal profiles is suggestive of some dissociation,but not as convincing as adding them both to a single model.

We appreciate this point. It is closely related to Reviewer 1's comment 2; please refer to our response above.

(2) The results for uncertainty and error show that uncertainty has strong effects before or at boundary onset, while error is related to more stabilization after boundary onset. This makes me wonder about the temporal contribution of each of these. Could it be the case that increases in uncertainty are early indicators of a boundary, and errors tend to occur later?

We also share the intuition that increases in uncertainty are early indicators of a boundary, and errors tend to occur later. If that is the case, we would expect some lags between prediction uncertainty and prediction error. We examined lagged correlation between prediction uncertainty and prediction error, and the optimal lag is 0 for both uncertainty-driven and error-driven models. This indicates that when prediction uncertainty rises, prediction error also simultaneously rises.

**Author response image 1.**

(3) Given that there is a 24-second period during which the neural responses are shaped by event boundaries, it would be important to know more about the average distance between boundaries and the variability of this distance. This will help establish whether the FIR model can properly capture a return to baseline.

We have added details about the distribution of event lengths. Specifically, we now report that the mean length of subjectively identified events was 21.4 seconds (median 22.2 s, SD 16.1 s). For model-derived boundaries, the average event lengths were 28.96 seconds for the uncertainty-driven model and 24.7 seconds for the error-driven model.

" For each activity, a separate group of 30 participants had previously segmented each movie to identify fine-grained event boundaries (Bezdek et al., 2022). The mean event length was 21.4 s (median 22.2 s, SD 16.1 s). Mean event lengths for uncertainty-driven model and error-driven model were 28.96s, and 24.7s, respectively (Nguyen et al., 2024)."

(4) Given that there is an early onset and long-lasting response of the brain to these event boundaries, I wonder what causes this. Is it the case that uncertainty or errors already increase at 12 seconds before the boundaries occur? Or if there are other makers in the movie that the brain can use to foreshadow an event boundary? And if uncertainty or errors do increase already 12 seconds before an event boundary, do you see a similar neural response at moments with similar levels of error or uncertainty, which are not followed by a boundary? This would reveal whether the neural activity patterns are specific to event boundaries or whether these are general markers of error and uncertainty.

We appreciate this point; it is similar to reviewer 2’s comment 2. Please see our response to that comment above.

(5) It is known that different brain regions have different delays of their BOLD response. Could these delays contribute to the propagation of the neural activity across different brain areas in this study?

Our analyses use ±20 s FIR windows, and the key effects we report include shifts ~12s before boundaries in higher-order cortex and ~4.5s pre-boundary in dorsal attention/parietal areas. Given the literature above, region-dependent BOLD delays are much smaller (~1–2s) than the temporal structure we observe (Taylor et al., 2018), making it unlikely that HRF lag alone explains our multi-second, region-specific progression.

(6) In the FIR plots, timepoints -12, 0, and 12 are shown. These long intervals preclude an understanding of the full temporal progression of these effects.

For page length purposes, we did not include all timepoints. We uploaded a brain animation of all timepoints and coefficients for each parcel in Openneuro (PATTERN_coefficients_brain_animation_human_fine_pattern.html and PATTERN_coefficients_lines_human_fine.html in the derivatives/figures/brain_maps_and_timecourses/ directory; https://doi.org/10.18112/openneuro.ds005551.v1.0.4) for interested researchers.

References

Taylor, A. J., Kim, J. H., & Ress, D. (2018). Characterization of the hemodynamic response function across the majority of human cerebral cortex. NeuroImage, 173, 322–331. https://doi.org/10.1016/j.neuroimage.2018.02.061
